# Emergence of apospory and bypass of meiosis via apomixis after sexual hybridisation and polyploidisation

**DOI:** 10.1111/nph.12954

**Published:** 2014-07-31

**Authors:** Diego Hojsgaard, Johann Greilhuber, Marco Pellino, Ovidiu Paun, Timothy F Sharbel, Elvira Hörandl

**Affiliations:** 1 Department of Systematics, Biodiversity and Evolution of Plants, Albrecht-von-Haller Institute for Plant Sciences, Georg August University of GöttingenUntere Karspüle 2, D-37073, Göttingen, Germany; 2 Division of Systematic and Evolutionary Botany, Department of Botany and Biodiversity Research, University of ViennaRennweg 14, A-1030, Vienna, Austria; 3 Apomixis Research Group, Leibniz Institute of Plant Genetics and Crop Plant ResearchCorrensstraβe 3, D-06466, Gatersleben, Germany

**Keywords:** allopolyploidy, aposporous initial cells, developmental biology, endosperm balance, megaspore functionality, ovule abortion, *Ranunculus*, sexuality

## Abstract

- Hybridisation and polyploidy are major forces contributing to plant speciation. Homoploid (2x) and heteroploid (3x) hybrids, however, represent critical stages for evolution due to disturbed meiosis and reduced fertility. Apomixis – asexual reproduction via seeds – can overcome hybrid sterility, but requires several concerted alterations of developmental pathways to result in functional seed formation.
- Here, we analyse the reproductive behaviours of homo- and heteroploid synthetic hybrids from crosses between sexual diploid and tetraploid *Ranunculus auricomus* species to test the hypothesis that developmental asynchrony in hybrids triggers the shift to apomictic reproduction.
- Evaluation of male and female gametophyte development, viability and functionality of gametes shows developmental asynchrony, whereas seed set and germinability indicate reduced fitness in synthetic hybrids compared to sexual parents. We present the first experimental evidence for spontaneous apospory in most hybrids as an alternative pathway to meiosis, and the appearance of functional apomictic seeds in triploids. Bypassing meiosis permits these triploid genotypes to form viable seed and new polyploid progeny.
- Asynchronous development causes reduced sexual seed set and emergence of apospory in synthetic *Ranunculus* hybrids. Apomixis is functional in triploids and associated with drastic meiotic abnormalities. Selection acts to stabilise developmental patterns and to tolerate endosperm dosage balance shifts which facilitates successful seed set and establishment of apomictic lineages.

## Introduction

Hybridisation and polyploidisation have long been recognised as motors of speciation and evolution in plants (Stebbins, 1950; Grant, 1981; Soltis & Soltis, 2009; Jiao *et al.* 2011). Reduced fertility due to meiotic disturbances, however, especially in the F_1_ generation, has led traditionally to a general perception of hybridisation as maladaptive (e.g. Arnold, 1997). Polyploidisation of hybrids (allopolyploidy) potentially can stabilise meiosis via homologous pairing of duplicated chromosomes (Comai, 2005). Natural polyploids (i.e. individuals having more than two haploid sets of chromosomes or genomes), which are common in most angiosperm families (Grant, 1981), can spontaneously arise multiple times in diploid populations by unilateral or bilateral sexual polyploidisation (Bretagnolle & Thompson, 1995). Unilateral sexual polyploidisation is a recurrent mechanism in nature associated with unreduced gamete formation (Bretagnolle & Thompson, 1995; Ramsey & Schemske, 1998; Mallet, 2007). Via the fusion of reduced gametes from one parent and unreduced ones from the other, triploid offspring can be formed. Triploid individuals, however, often have strongly reduced fitness because of meiotic disturbances and low fertility. Nonetheless, even low frequencies of unreduced gamete formation may allow for the generation of cytologically more stable tetraploids (Ramsey & Schemske, 1998). This so-called ‘triploid bridge’ is regarded as a major route to plant polyploidisation and speciation (Ramsey & Schemske, 1998; Rieseberg & Willis, 2007). In this frame, the way(s) diploids but mainly triploid hybrids tackle infertility due to uneven chromosome numbers, meiotic irregularities and/or dosage balances of male-to-female contributions to seed formation are crucial determinants of their reproductive and surviving success.

Shifts to apomixis via asexual seed formation in plants (agamospermy) have long been claimed as a possible mechanism to escape from hybrid sterility and to stabilise polyploid hybrid biotypes (e.g. Stebbins, 1950; Grant, 1981; Asker & Jerling, 1992). In apomicts, the bypass of meiosis for embryo sac development preserves the female genetic constitution, and allows for the maintenance of highly heterozygous hybrid biotypes. Evidence for hybrid origin of natural apomictic taxa is available in an increasing number of molecular studies (e.g. Koch *et al.* 2003; Paun *et al.* 2006a; Fehrer *et al.* 2007; Kantama *et al.* 2007). Nevertheless, it is still not fully understood how hybridisation and/or polyploidisation actually trigger and establish apomictic reproduction in natural populations.

Molecular studies on the genetic control of apomixis suggest that the phenomenon is caused by an epigenetic temporal or spatial deregulation of the sexual system rather than an independent trait (e.g. Carman, 1997; Grimanelli *et al.* 2003; Koltunow & Grossniklaus, 2003; Curtis & Grossniklaus, 2008; Grimanelli, 2012). Heterochronic shifts during sporogenesis and gametogenesis are supposed to suppress the sexual pathway in favour of apomictic development (Carman, 1997; Sharbel *et al.* 2010). Apomixis involves three alterations of female development (Asker & Jerling, 1992). First, an unreduced megaspore-like cell is formed by bypassing meiosis to undergo gametogenesis and to develop into a female gametophyte (embryo sac). Second, the unreduced egg cell develops into an embryo without fertilisation (parthenogenesis). Third, in most cases a male germ cell must fertilise the central cell of the female gametophyte for proper formation of the endosperm, the nutritious tissue of the embryo (pseudogamy) (e.g. Koltunow & Grossniklaus, 2003). The latter is a constraint of genomic imprinting in the endosperm which requires the male function (Vinkenoog *et al.* 2003). For this reason, male meiosis and sporogenesis need to proceed without major alterations for pollen to be functional. Thus, the shift to apomixis requires several steps, each of it alone being deleterious; apomeiosis without parthenogenesis leads to a continuous increase of ploidy levels, parthenogenesis alone results in haploid plants in which deleterious recessive alleles are fully expressed. Natural selection will therefore act against single components of apomixis: only the combination of such features will be successful (van Dijk & Vijverberg, 2005; Hörandl, 2009).

The *Ranunculus auricomus* complex comprises diploid and tetraploid sexual species (*R. carpaticola*, *R. notabilis* and *R. cassubicifolius*) and many apomictic polyploids, among them the apomictic allohexaploid *R. carpaticola* × *R. cassubicifolius* (Paun *et al.* 2006a; Hörandl *et al.*, 2009). In *Ranunculus*, as in many angiosperms, allopolyploidy is frequently linked to shifts in reproductive traits such as self-compatibility and apomictic reproduction (Comai, 2005; Barringer, 2007; Robertson *et al.* 2010; Pellino *et al.* 2013). In *Ranunculus auricomus*, a somatic cell of the nucellus develops into an unreduced embryo sac (apospory), whereas meiotic products abort. The egg cell with a somatic chromosomal constitution develops parthenogenetically, but pseudogamy is essential for seed development (Nogler, 1984). Sexual diploid and tetraploid species are self-incompatible, while polyploid apomicts are self-fertile (Hörandl, 2008). Nevertheless, so far the occurrence and/or the role of reproductive shifts in newly formed hybrids have not yet been explored.

The aim of this study was to screen for shifts in modes of reproduction in homoploid and heteroploid hybrid plants of the *Ranunculus auricomus* complex. Because relative divergence between parental genomes has an influence on allopolyploid formation (Paun *et al.* 2009), and considering that natural apomicts of the *R. auricomus* complex are putative allopolyploid hybrids (Paun *et al.* 2006a), we expected to detect spontaneous shifts in the reproductive biology of interspecific, rather than of intraspecific crosses. To examine effects of polyploidisation, we use an experimental approach to form both diploid and triploid hybrid individuals, followed by analyses of changes in reproductive pathways. According to phylogenetic relationships within the genus (Hörandl *et al.*, 2005; Hörandl & Emadzade, 2012) we chose as parents the diploid sexual species *R. notabilis* – as the most distantly related taxon – and the sister species pair *R. carpaticola* (2x) and *R. cassubicifolius* (4x). By crossing diploid (donors of haploid gametes) and autotetraploid (donors of diploid gametes) sexual *Ranunculus* species, we produced diploid (between homoploid parentals) and triploid (between heteroploid parentals) hybrids. We consider both cases as synthetic individuals that mirror naturally occurring hybrids. We screened the offspring for the spontaneous appearance of apomixis-like phenomena at the sporogenesis, gametogenesis and embryogenesis (seed formation) stages, and analysed the effects of hybridity and ploidy on such reproductive shifts. We carefully monitored the timing of sporogenesis and gametogenesis with respect to ovule rotation to test the hypothesis that apomixis is connected to an asynchrony of developmental steps. We finally consider the potential consequences of such reproductive shifts on endosperm formation, seed set and the subsequent evolutionary establishment of apomictic lineages. The closely related naturally occurring apomictic allohexaploid (*c*. 60 000–80 000 yr old; Pellino *et al.* 2013) and parental genotypes were used as reference examples to better understand the function of apomixis in polyploid hybrid evolution.

## Materials and Methods

### Plant materials

Natural plants from diploid sexual allogamous *Ranunculus carpaticola* Soó and *R. notabilis* Hörandl & Guterm., tetraploid sexual allogamous *R. cassubicifolius* W. Koch, and hexaploid autogamous apomictic *R. carpaticola* × *R*. *cassubicifolius* (Hörandl & Greilhuber, 2002; Paun *et al.* 2006a; Hörandl, 2008) were selected from materials collected in wild populations (Supporting Information Table S1) and are grown in the experimental garden at the Albrecht-von-Haller Institute for Plant Sciences, Faculty of Biology and Psychology, Georg August University of Göttingen, Germany.

In order to evaluate reproductive capacities, experimental homo- and heteroploid hybrids were obtained during the summer of 2006 after controlled handmade pollinations in emasculated maternal flowers between diploid *R. carpaticola* × diploid *R. notabilis* and tetraploid *R. cassubicifolius* × diploid *R. notabilis* (Table S1). Parental taxa represent phylogenetically and morphologically distinct species within the genus (Hörandl & Emadzade, 2012). Because of potential parent-of-origin effects causing ovule abortion and lower fertility in interploidal crosses (e.g. Lin, 1984; Scott *et al.* 1998), excess in paternal genome contributions were avoided by using diploid *R. notabilis* as pollen donor in all cases. Seeds were potted in sterilised soil and grown under the same light and water regime. Hybrid individuals are cultivated in the experimental garden.

### Determination of hybridity in synthetic individuals

The morphotype of synthetic hybrids was not intermediate but strongly resembled that of the pollen parent, *R. notabilis* (Hodač *et al.*, 2014). In addition, the hybrid nature of the experimental progeny was ascertained by ploidy level and molecular marker segregation analyses.

#### DNA content and ploidies

The level of ploidy was determined through genome size evaluation and chromosome counting. Genome size on parental genotypes and F_1_ descendants from experimental crosses was estimated by Feulgen DNA image densitometry according to Greilhuber & Temsch (2001). Root tip cells from parental and putative hybrids were fixed in ethanol : acetic acid (3 : 1) for quantitative Feulgen staining (Schiff′s reagent, C_20_H_21_N_3_SO_3_). Relative nuclear DNA content was calculated from an average of 50 Feulgen-stained nuclei, as proportional to *Pisum sativum* L. (‘Kleine Rheinländerin’; 2C = 8.84 pg) internal standard.

The chromosome number was checked in mitotic cells of 10 randomly selected F_1_ individuals, five diploids and five triploids. Actively growing root tips were treated with 8-hydroxiquinoline for 6.5 h (3 h at RT and 3.5 h at 5°C), fixed for 24–48 h in ethanol : acetic acid (3 : 1), hydrolysed for 10 min in 1 N HCl at 60°C, and stained with Schiff′s reagent. The chromosome number of at least five good metaphase cells per plant was examined with an Axio Imager.A1 microscope (Carl Zeiss GmbH, Jena, Germany). Images were taken with AxioVision 4.8 software (Carl Zeiss GmbH) and corrected for contrast with Adobe® Photoshop CS4 (Adobe Systems Software Ireland Ltd, Dublin, Ireland).

#### Genetic markers

A sample of 60 out of 4299 primer pairs for microsatellites (SSR) collected using SciRoKo software (Kofler *et al.* 2007) from genomic cDNA sequences (Pellino *et al.* 2013) were selected and tested in a pre-analysis using samples of each parent and bulked progenies. PCR protocols for microsatellite analysis followed Cosendai *et al*. (2011), and microsatellite amplicons were first screened on 1.5% agarose gel electrophoresed 2 h at 60 V. Two informative SSR primer pairs (*Rau*10_CTTTGGGCTACCAGCATAGG/GGAAGGATCATGCTAAGGCA and *Rau*16_AACCCGACCACAAATGAGAG/TTCTGATGACATCCGTGGAA) revealing polymorphic fragments between parental genotypes and paternal inheritance in synthetic hybrids were then sliced, DNA fragments were recovered using Invisorb® Fragment CleanUp (STRATEC Biomedical AG, Birkenfeld, Germany), cleaned using Sephadex G-50 Superfine (GE Healthcare Bio-Sciences, Uppsala, Sweden) following manufacturer's instructions, and sequenced using BigDye Terminator RR mix (Applied Biosystems, Foster City, CA, USA) and an ABI Prism 377 DNA sequencer (Perkin-Elmer, Norwalk, CT, USA). Sequence analyses were carried out in Geneious software (Biomatters, Auckland, New Zealand).

### Male and female sporogenesis and gametogenesis

Flower buds of wild sexual, apomictic and synthetic plants in different developmental stages were fixed in formalin : acetic acid : ethanol : dH_2_O (2 : 1 : 10 : 3.5) (FAA) for 48 h, and stored in 70% ethanol at RT. The perianth of selected flowers and flower buds was removed, and gynoecium plus 6–10 stamens were cleared in methylsalicylate (Merck KGaA, Darmstadt, Germany), using the procedure outlined by Young *et al*. (1979) with minor modifications. Materials were dehydrated by four steps of 30 min incubation in 1 ml of 70%, 95% and 100% ethanol (two times). Then, materials were steeped for 30 min in 300 μl of upgrading series of methylsalicylate diluted in ethanol (25%, 50%, 70%, 85% and 100%). Ovaries (pistils) and anthers were dissected, mounted on glass slides, and female sporogenesis and gamete development analysed with a Zeiss Axiophot microscope with Nomarski DIC optics (Carl Zeiss GmbH). Different stages of gametogenesis and sporogenesis were evaluated in at least 75 cleared ovules and stamens per genotype, in eight sexual, 10 apomictic and 24 hybrid genotypes, including 15 synthetic diploids and nine triploids. As both *Polygonum* and *Hieracium* types of development are structurally and anatomically similar, the proportions of sexuality/apomixis were estimated at the end of sporogenesis/beginning of gametogenesis.

### Ovule morphometrics

Ovule rotation in ovaries was measured at the end of sporogenesis and gametogenesis in four sexual, six apomictic genotypes and 14 synthetic hybrids (eight diploids and six triploids) (Table S2). Mature ovules in *Ranunculus* are anatropous. Similar to *Arabidopsis* (e.g. Sieber *et al.* 2004), the ovule primordium in *Ranunculus* develops perpendicular to the basal gynoecium and creates a Proximal–Distal (P–D) axis. Meanwhile, the germline goes through sporogenesis and gametogenesis, and an asymmetric growth bends the P–D axis and creates an Anterior–Posterior (A–P) axis. Hence, ovule curvature was measured on this A–P axis as an external timing marker to reproductive stages, to pursue the progression of reproductive pathways (see Fig. S1). Data were taken using a digital image analysis system (AxioVision 4.8.1; Carl Zeiss GmbH). The two key developmental stages were evaluated and all statistical analyses were performed with the SPSS® statistical program (IBM, Armonk, NY, USA).

### Male gametophyte stainability and pollen tube growth

Pollen stainability and pollen tube elongation were further analysed in a sample of 21 individuals to corroborate male gamete viability and functionality among diploid and triploid hybrids. As a reference, six parental genotypes and six natural hybrids were analysed. Pollen stainability was assessed on at least 200 grains per genotype, by 1% Lugol's iodine (I_2_KI; Merck KGaA). Lugol's detection of starch and intact cell membranes provides indirect evidence of viability (Dafni & Firmage, 2000). A total of 17 512 pollen grains were classified as viable or nonviable according to the presence of amylose and amylopectin polysaccharides (Table S3). Pollen tube growth analysis on four parental genotypes, six natural hybrids and ten synthetic hybrids followed Kho & Baer (1968). Flowers of crossed genotypes of *R. notabilis*, *R. cassubicifolius*, diploid and triploid hybrids, and self-pollinated hexaploid apomictic genotypes were fixed in FAA after 30, 60, 120 and 180 min post-pollination (Table S4). Pollen tubes in pistils were detected in complete pistils using a Leica DM5500B microscope with an UV lamp and a long-pass filter cube for blue (425 nm). Images were acquired with a DC450 camera (Leica Microsystems GmbH, Wetzlar, Germany).

### Seed set and reproductive pathway assessment

Seed production among ploidies and species was evaluated after bagging open and/or hand-pollinated flowers according to Hörandl (2008). Single seeds were manually chopped with a new razor blade in a plastic petri dish (60 mm) in 250 μl isolation buffer (0.1 M citric acid monohydrate and 0.5% v/v Tween 20 (Sigma, St Louis, MO, USA) adjusted to pH 2.5), the suspension was filtered (30-μm mesh CellTrick® filters; Partec GmbH, Münster, Germany), stained with 1.0 ml of staining buffer (0.4 M Na_2_HPO_4_ dissolved in H_2_O plus 4 μg ml^−1^ DAPI (C_16_H_15_N_5_), adjusted to pH 8.5 (Otto, 1990)), incubated for 10–15 min on ice, and analysed on the manual sample port of a Partec PAII flow cytometer (Partec GmbH). Furthermore, a high-throughput automated method was used, in which one to two seeds were ground at a rate of 300 strokes min^−1^ for 90 s (using a Geno-Grinder 2000; SPEX CertiPrep, Metuchen, NJ, USA) in a 96 deep well plate (PP-Masterblock 128.0/85 mm, 1.0 ml 96-well plate; Greiner Bio-One, Frickenhausen, Germany) with three 2.3-mm stainless steel beads and 50 μl isolation buffer in each well. After grinding, an additional 250 μl buffer was added, and the solution filtered (30-μm mesh CellTrick® filters). A 50-μl volume of the suspension was mixed with 0.1 ml of staining buffer, incubated on ice for 10 min and analysed by an automated flow cytometer. In both the manual and the automated approaches, the fluorescence intensity of DAPI-stained nuclei was determined at 355 nm. About 3000 nuclei were measured in manually chopped samples, and at least 1000 nuclei in automated samples. All histograms were analysed using PA II's Partec FloMax® software (Partec GmbH). The coefficient of variation was 8% or less. Diploid *R. notabilis* and *R. carpaticola*, or tetraploid *R. cassubicifolius* were used as references for ploidy analyses of embryo and endosperm tissues. Functionality of apomixis components and reproductive pathways were recognised following the rationale of Matzk *et al*. (2000) and Hojsgaard *et al*. (2013). After fertilisation, sexually derived (haploid) embryo sacs will develop a diploid embryo (2*n*, by fusion of 1 maternal + 1 paternal genomes; 2*C*-value) and a triploid endosperm (3*n*, by fusion of 2 maternal + 1 paternal genomes; 3*C*-value). In apomictic (diploid) embryo sacs, the embryo develops from the egg cell by parthenogenesis (2*n*, 2 maternal genomes; 2*C*-value) but the unreduced nuclei (2*n* each) of the central cell are fertilised to form the endosperm (5*n*, by fusion of 4 maternal + 1 paternal genomes; 5*C*-value). In some cases, two sperm nuclei can contribute to form the endosperm (6*n*, by fusion of 4 maternal + 2 paternal genomes; 6*C*-value). The relative proportions of viable gamete formation were calculated from the number of developed seeds.

### Germinability and ploidy levels in F_2_ individuals

In order to test for fertility of triploids and ploidy variation in their offspring, triploid × triploid crosses were done and a sample of seeds sown in sterilised soil, grown with same conditions of relative humidity, temperature and light in a glasshouse at the Georg August University of Göttingen Botanical Garden. Ploidies of seedlings were determined by chromosome counting as previously specified.

## Results

### Experimental hybridisation events among sexual species

A total of 82 F_1_ plants arising from experimental crossings between sexuals were successfully grown in the experimental garden. Fifty individuals were derived from *R. notabilis* (2x) × *R. carpaticola* (2x), whereas 32 originated from *R. notabilis* (2x) × *R. cassubicifolius* (4x). Genome size analyses in hybrids confirmed the expected diploidy for *R. carpaticola* × *R. notabilis* and triploidy for *R. cassubicifolius* × *R. notabilis* progenies (Table S1; Fig. S2a). Chromosome numbers in mitotic cells of sampled F_1_ individuals furthermore corresponded to diploid (2*n* = 2x = 16) and triploid (2*n* = 3x = 24) genomes (Fig. S2b). Triploidy in F_1_ descendant plants from diploid × tetraploid crosses prove the hybrid nature of these genotypes, considering the fusion of two meiotically reduced parental gametes (x + 2x), as do the presence of two paternally inherited microsatellite alleles which were found in all F_1_ plants (e.g. *Rau*10 SSR in Fig. S2c).

### Female and male sporogenesis and gametogenesis in parental species

Overall, 419 ovules and 73 anthers were analysed at different developmental stages throughout megaspore mother cell (MMC) and pollen mother cell (PMC) formation, meiosis and gametogenesis in diploid *R. carpaticola* and *R. notabilis* and tetraploid *R. cassubicifolius*. Obligate sexuality was confirmed in both diploid and tetraploid parental species and support previous population genetic and pollen viability data (Hörandl *et al.*, 1997, 2000; Paun *et al.* 2006b). The main features of female ovule development in sexuals include: (1) regular sporogenesis forming a dyad after the first (reductional) division, and a triad (sometimes a tetrad) due to absence of cytokinesis in the micropylar dyad during the second (equational) division (Fig. S1a); (2) subsequent selection of the chalazal megaspore and abortion of the others while the ovule was *c*. 90° rotated on its A–P axis (Figs S1b, S3a, Table S2); and (3) *Polygonum*-type gametophyte development of the functional megaspore along ovule axis rotation till anatropous placement of the ovule (*c*. 165° rotated on its A–P axis), and formation of a heptanucleated and seven-celled embryo sac (Figs S1c, S3c, Table S2). Nuclei redistribution and fusion inside pre-mature eight-nuclei vacuolated female gametophytes took place simultaneously with cellularisation and cell differentiation just before flowering. In one out of 26 ovaries of *R. carpaticola* (genotype Hö8483; Table S1) analysed, an ovule with two embryo sacs was observed at flowering.

Male sporogenesis was normal, first and second meiotic divisions were regular, and simultaneous-type cytokinesis produced four microspores from each pollen mother cell. Mature male gametophytes were binucleated and two-celled (one generative cell and one tube cell) during flowering, after a post-meiotic mitosis on uninucleated microsporocytes. Male gametophyte viability evaluated in 1773 pollen grains from three parental genotypes (Table S3) showed 77.7% viable microgametophytes, corroborating previous analyses (Hörandl *et al.*, 1997). Parental species are self-incompatible, and cross-pollinated flowers produced sexually derived seeds (the ‘seed formation’ section below).

### Alterations of female and male sporogenesis and gametogenesis and emergence of aposporous-like cells in synthetic hybrids

**Fig 1:**
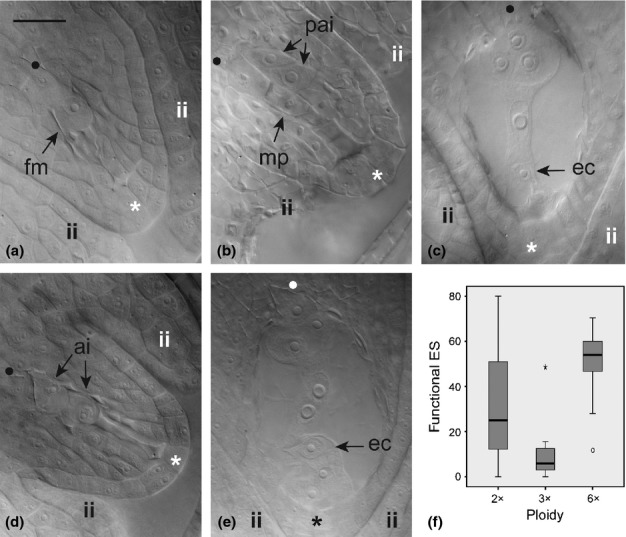
Key female gametophyte reproductive stages during flower development in 2x homoploid and 3x heteroploid synthetic hybrids (a–c), and natural 6x heteroploid hybrids (d, e) of *Ranunculus*. Ovule images are placed as in Supporting Information Fig. S1 so that rotation angles can be tracked by the orientation of the chalazal-micropylar axis. (a) Functional megaspore immediately after meiosis; (b) end of meiosis showing four meiotic products – the two located toward the micropyle are aborted whereas the other two show signs of abortion – and two putative aposporous initial cells; (c) completely rotated ovule showing a mature seven-celled embryo sac at blooming stage; (d) aborted germline and two aposporous initials in the chalazal area; (e) mature embryo sac just before polar nuclei fusion to form a secondary nucleus in the central cell. (f) Box-and-whisker diagram for the proportion of functional embryo sacs (ES) among ploidy levels of interspecific hybrids. Boxes represent first and third quartiles, and the band inside each box indicates the median. Whiskers correspond to 95% CI. Outliers and extreme values are represented by circles and stars, respectively. Genotypes: (a) J5; (b) J20; (c) G13; (d) HöC29; (e) HöC35. fm, functional megaspore; mp, row of four meiotic products; pai, putative aposporous initial cells; ec, egg cell; ai, aposporous initial cells; ii, inner integuments; •, chalazal pole; *, micropylar pole. Bar, 30 μm.

Ovule development in diploid (*R. carpaticola* × *R. notabilis*) and triploid (*R. cassubicifolius* × *R. notabilis*) hybrids (*n *=* *910; Table 1) was structurally similar to that of sexual parents, but showed clear alterations in timing, and bias in the reproductive mode. Once started, sporogenesis and gametogenesis were delayed compared to parental species. At the end of meiosis, ovules were rotated *c*. 150° on the A–P axis in both diploid and triploid hybrids (Figs1a, S3d, Table S2), and a greater variance in rotation angles at this stage evidenced meiotic developmental asynchrony (Table S2). Megasporogenesis exhibited diverse developmental irregularities (e.g. arrested development and apoptosis in germ line cells at different points of meiosis, altered pattern of megaspore selection; Fig. S4a,b). When functional, meiosis ended in a triad (Fig. 1a), although dyads or tetrads were often observed. Functional megaspores were present in 67% of diploid ovules (*n *=* *257; Table 1) and 69% of triploid ovules (*n *=* *191; Table 1). At this stage, 11–15% of ovules showed enlarged somatic cells (i.e. putative aposporous initial or aposporous initial-like cells) in the nucellar tissue of diploids and triploids, respectively (Fig. 1b; Table 1). Putative aposporous cells appeared during meiosis, when apoptosis in meiotic germline was evident, or after meiosis at the 1-to-4 nuclei embryo sac stage.

**Table 1:** Proportion of ovules showing a functional germline and/or aposporous-like activity in the surrounding somatic tissue at the end of sporogenesis and gametogenesis in *Ranunculus* species and hybrids of different ploidy and origin

Taxa	*n*	Sporogenesis			*n*	Gametogenesis	
		Fm (range)	pAI (range)	Abov (range)		MfG^1^ (range)	Ab/uG^2^ (range)
Parentals							
* R. notabilis* 2x	86	0.96 (0.94–1.00)	0.0	0.04 (0.0–0.05)	66	0.96 (0.88–1.0)	0.04 (0.00–0.12)
* R. carpaticola* 2x	135	0.84 (0.83–0.90)	0.0	0.16 (0.10–0.18)	89	0.95 (0.93–0.96)	0.05 (0.00–0.06)
* R. cassubicifolius* 4x	98	0.95 (0.94–1.00)	0.0	0.05 (0.00–0.06)	103	0.94 (0.85–0.96)	0.06 (0.00–0.08)
Synthetic hybrids							
* R. carpaticola* × *R. notabilis* 2x	257	0.67 (0.44–1.00)	0.11 (0.00–0.33)	0.22 (0.00–0.56)	289	0.41 (0.08–0.80)	0.59 (0.20–0.92)
* R. cassubicifolius* × *R. notabilis* 3x	191	0.69 (0.54–0.87)	0.15 (0.07–0.32)	0.15 (0.00–0.29)	173	0.21 (0.00–0.48)	0.79 (0.48–1.00)
Natural hybrids							
* R. carpaticola* × *R. cassubicifolius* 6x	218	0.69 (0.56–0.96)	0.67 (0.50–0.87)	0.07 (0.00–0.13)	285	0.48 (0.17–0.75)	0.52 (0.25–0.81)

*n*, number of ovules analysed; Fm, functional megaspores; pAI, putative aposporous initial cells; Abov, aborted ovules; MfG, mature female gametophytes; Ab/uG, aborted/undeveloped gametophytes.

1 Embryo sacs with 8N just before cellularisation process were considered as mature.

2 See details about classes of undeveloped gametophytes in the text.

Gametogenesis in the meiotic functional megaspore followed the pattern observed in parental species, and three mitoses produced one polarised, heptanucleated seven-celled mature embryo sac (Fig. 1c). However, like megasporogenesis, succeeding megagametogenesis showed a variety of developmental irregularities among genotypes (e.g. arrested embryo sac development, uneven nuclear migration during embryo sac polarisation, abortion of embryo sac nuclei at the micropylar pole; Fig. S4c–e), encompassing the suppression of the meiotic pathway. Simultaneously to the meiotic pathway, gametogenesis proceeded in some putative aposporous initial cells of the somatic tissue, with subsequent development of a chromosomally unreduced megagametophyte structurally similar to those derived meiotically – that is, seven differentiated cells but with unreduced nuclei. As a result of altered sporogenesis and gametogenesis, the proportions of mature female gametes varied among synthetic genotypes and ploidy levels (see Fig. 1f, Table 1). Most anatropous fully grown ovules (on average 165° rotated on the A–P axis; Fig. S3e, Table S1) showed no mature embryo sacs but went through other developmental stages by the end of gametogenesis. Thus, almost 60% of diploid ovaries (*n *=* *289; Table 1) and 80% of triploid ovaries (*n *=* *173; Table 1) analysed at flowering had one functional – or aborted – megaspore (i.e. arrested and/or suppressed gametogenesis), 1–4 nucleated embryo sacs (i.e. delayed gametogenesis), embryo sacs with eight undifferentiated nuclei or no embryo sac at all (i.e. suppressed and/or delayed cellularisation, aborted development) (Fig. S4c–e, Table 1).

**Fig 2:**
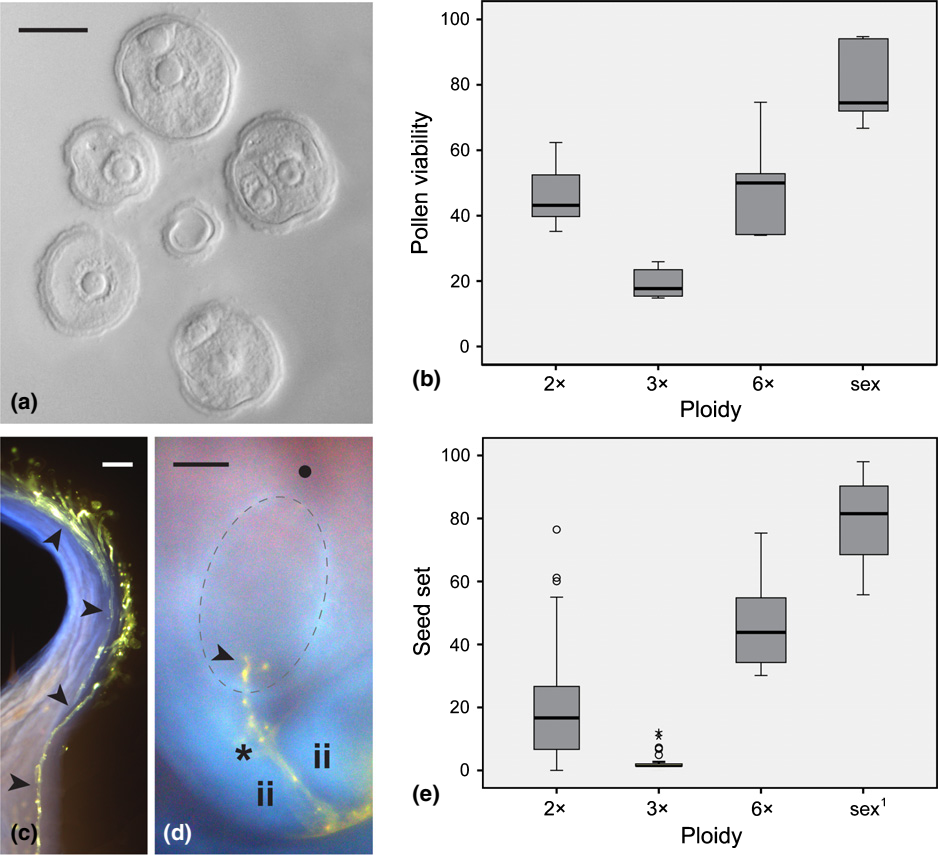
Male gametophyte development, viability, functionality and compatibility with female gametophytes to set seeds in *Ranunculus*. (a) Male gametophytes of a triploid hybrid during flowering showing aborted and immature to mature microspores (carrying one nucleus or two, with the generative one adsorbed to the inner microspore wall). (b) Box-and-whisker diagram for the proportion of viable male gametophytes at flowering among ploidy levels of interspecific hybrids and sexual parentals. Boxes represent first and third quartiles, and the band inside each box indicates the median. Whiskers correspond to 95% CI. Outliers and extreme values are represented by circles and stars, respectively. (c) Growing pollen tubes of functional male gametophytes on flower stigma and style (arrowheads) 120 min post-pollination; (d) ovule at flowering, 180 min post-pollination, showing the path of the pollen tube toward the embryo sac (whitish delineated) between integuments and the apparent delivery of male gametes into the synergids and egg cell zone (arrow). (e) Box-and-whisker diagram for the proportion of seeds formed by interspecific hybrids and sexual parental species among ploidy levels. Boxes, whiskers and outliers are represented as in (b). Genotypes: (a) G4; (c) G7; (d) J33. ii, inner integuments; •, chalazal pole; *, micropylar pole. Bars: (a) 10 μm; (c, d) 30 μm. ^1^Data from Hörandl (2008).

Male meiosis was apparently regular in synthetic diploids, but less than a half of mature microgametophytes (45.3%, ranging between 32.3% and 62.4%, Table S3) had polysaccharide vesicles in their cytoplasm, indicating viability. In triploids, male meiosis was clearly irregular: the progress through microsporogenesis was disturbed from the beginning and showed high amounts of cell abortion, ending with an average of < 20% viable microgametophytes (ranging between 10.6% and 25.9%; Fig. 2a,b, Table S3).

### Female and male sporogenesis and gametogenesis in natural apomictic hybrid genotypes

In comparison, female sporogenesis and gametogenesis in natural hexaploid *R. carpaticola* × *R. cassubicifolius* hybrids showed intermediate characteristics, as the development was delayed in timing and functionally altered compared to parental sexual genotypes, but it was not delayed as much and was functionally more stable than in synthetic hybrids.

At the end of meiosis, the chalazal megaspore remained functional in 69% of ovules (*n *=* *218) which were rotated 120° on its A–P axis (Fig. S3c, Tables1, S1). Regardless of the functionality of the megaspore, a high proportion of ovules showed one to several actively growing somatic cells in the nucellar tissue (67%, *n *=* *218; Fig. 1d, Table 1). Precocious development of these aposporous cells was apparent in some ovules before they attained a 90° rotation on the A–P axis, displaying the presence of aposporous cells or 1-to-2 nuclei embryo sacs before female meiosis finished. In a few ovules (3.6%, *n *=* *83; not shown), both aposporous and meiotic embryo sacs developed simultaneously until the first or second mitotic divisions of gametogenesis (2-to-4 nuclei embryo sacs). Gametogenesis in meiotic (reduced) and somatic (nonreduced) pathways was structurally similar to that observed in sexual parentals, and one mature embryo sac (*Polygonum*- or *Hieracium*-type) was observed at anthesis in 48% of ovules (*n *=* *285; Fig. 1f, Table 1; final rotation angle of ovules *c*. 170° on the A–P axis; Fig. S3e,f, Table 1). The other half of ovules showed underdeveloped embryo sacs, including 6.7–16% ovules with no embryo sac (Table 1).

**Fig 3:**
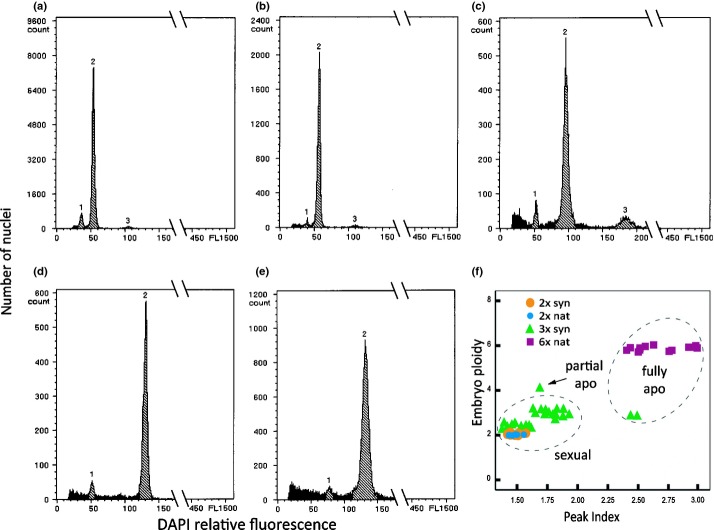
Flow cytometry histograms of seeds produced by diploid *Ranunculus carpaticola* × *R. notabilis* (a) and triploid *R. cassubicifolius* × *R. notabilis* hybrids (b–e). In all histograms: peak 1, nuclei from the embryo tissue; peak 2, nuclei from the endosperm tissue; peak 3, dividing cells of the embryo tissue in G_2_ phase of the mitotic cycle. (a) Sexual seed from a diploid mother plant with a diploid embryo (*c*. 35 in *x*-axis) and a triploid endosperm (*c*. 50 in *x*-axis) formed by fertilisation of a reduced female gametophyte; (b) sexual seed from a triploid mother plant with a near diploid embryo and a near triploid endosperm formed after fusion of near haploid gametes; (c) sexual seed from a triploid plant, with a triploid embryo and a hexaploid endosperm formed by polyspermy; (d) apomictic seed from a triploid mother plant, with a triploid embryo formed by parthenogenesis and a near octoploid (*c*. 7.5x) endosperm formed by fertilisation of a hexaploid secondary polar nucleus derived from an unreduced central cell; (e) partial apomictic seed from a triploid plant, with a near tetraploid embryo (*c*. 4.4x) and a near heptaploid endosperm (*c*. 7.4x) formed by fertilisation of a hexaploid secondary polar nucleus derived from an unreduced central cell; (f) plot representing the variety of cytological pathways of seed formation observed in diploid and polyploid materials according to peak indexes and ploidy of embryos. syn, synthetic genotypes; nat, natural genotypes. Genotypes: (a) F11; (b) G6; (c) G12; (d) G1; (e) I9.

On the male side, meiosis occurred concurrently with ovule rotation, as observed in the sexual parental species. Differentiated microsporocytes, vacuolated with one nucleus and apparent cell wall, were observed in flower buds by the end of female meiosis. One post-meiotic mitosis formed the binucleated microsporocyte released during anthesis. The mean proportion of viable male gametes among genotypes was 53.2% pollen grains (Fig. 2b,Table S3).

### Megaspore functionality and ovule abortion in synthetic and natural hybrids

At the end of meiosis a strong correlation between the presence of putative aposporous initial cells and aborted functional megaspores was observed in ovules of diploid (*r *=* *0.93, *P *<* *0.01) and triploid (*r *=* *0.79, *P *<* *0.03) synthetic hybrids, as well as in the natural hexaploid hybrids (*r *=* *0.70, *P *<* *0.03). Similarly, Pearson product-moment correlation coefficients were significant when the presence of putative aposporous initial cells was compared to aborted ovules at flowering (i.e. absence of functional reproductive pathways) in diploids and triploids (*r *=* *0.74, *P *<* *0.01; and *r *=* *0.80, *P *<* *0.02, respectively). By contrast, no correlation was found in natural hexaploids (*r *<* *0.01, *P *=* *0.99), indicating that developmental alterations leading to abortion of female megaspores and aposporous expression are correlated with ovule abortion in synthetic but not in natural hybrids.

### Male gametophyte functionality in parental species, natural and synthetic hybrids

Two hundred and twenty-two post-pollinated pistils were analysed for pollen tube development (Table S4). The tube cell developed within first 30 min and reached the middle-end of the style and upper ovary within 60 min in sexual parentals, natural hexaploid hybrids and diploid synthetic hybrids (Fig. 2c, Table S4). After 120 min the pollen tube reached the micropylar area of the ovule in parental genotypes, but not in all pistils from natural and synthetic hybrids. Following 180 min post-pollination, at least one pollen tube was observed in the micropyle of all ovaries analysed, indicating that gametes were discharged in the area of the embryo sac where synergid cells are located (Fig. 2d, Table S4). In triploid hybrids, the number of pollen grains on stigma was drastically reduced as a direct consequence of low pollen viability (Table S4). Moreover, in at least two triploid genotypes (G7 and G20), activation of pollen tube development was delayed after 30 min post-pollination (no tube growth was observed), whereas after 60 min pollen tubes were barely penetrating the papillae cells. Despite this, pollen tube tips were observed at the micropyle of ovules after 180 min post-pollination (Table S4).

### Synthetic hybrids show sexual and apomictic seed formation

In natural hexaploid hybrids, 45.7% fully developed seeds were recovered on average (Fig. 2e, Table S5). In synthetic hybrids, seed set was *c*. 20% among 46 intercrossed combinations of F_1_ diploids *R. carpaticola* × *R. notabilis*, and considerable lesser (0.86%) among 29 intercrossed combinations of F_1_ triploids *R. cassubicifolius* × *R. notabilis* (Fig. 2e, Table S5).

In total, embryo and endosperm ploidies of 1396 seeds from three diploid parents, 10 natural hexaploid and 25 synthetic hybrids, representing 15 diploid and 10 triploid genotypes, were analysed by flow cytometry (Tables2, 3). Endosperm-to-embryo peak indexes revealed the nature of seed formation pathways. Around 29% of 734 seeds from hexaploid natural hybrids were sexually produced (peak indexes ranged *c*. 1.5–2; 2m : 1p contributions to the endosperm; Table 2). The remaining seeds showed peak indexes between *c*. 2.5 and 3 due to the participation of unreduced female gametophytes in zygote formation plus one or two sperms in endosperm formation (relative contributions of 4m : 1p and 2m : 1p to the endosperm, respectively; Tables2, 3). Natural diploid parents and newly formed diploid hybrids consistently produced sexual seeds (*n* = 30 and *n* = 501, respectively, 2m : 1p contributions to the endosperm; Tables2, 3) (Fig. 3a). However, triploid hybrids showed a variety of functional cytological pathways for seed formation. Analysis of reproductive pathways, ploidy ranges in embryos and parental contributions to endosperm (Tables2, 3) indicated that most seeds were sexually derived, with near diploid or near triploid embryos resulting from the fusion of near haploid or hyperhaploid male and female gametes carrying unbalanced aneuploid chromosome sets (unbalanced meiosis + syngamy; *n *=* *50; *c*. 2m : 1p contributions to the endosperm; Fig. 3b, Table 3). Some seeds carried triploid embryos (3x; *n *=* *8), of which three had near hexaploid endosperms (*c*. 6x) (Fig. 3c, Table 3), suggesting syngamy and polyspermy in the central cell involving either hyperhaploid female and male gametes (*c*. 1.5x), or a near diploid female gamete and near haploid male gametes (unbalanced meiosis + syngamy + polyspermy) (Fig. 3f; Tables2, 3). Two seeds with triploid embryos developed apomictically from an unreduced, unfertilised egg cell and a fertilised central cell (apospory, parthenogenesis + pseudogamy; 3x embryo, *c*. 7.5x endosperm; *c*. 4m : 1p contributions to the endosperm; Fig. 3d; Table 2). One seed had a near tetraploid embryo (*c*. 4.4x), which was formed after fertilisation of an unreduced embryo sac by two reduced male gametes (*c*. 1.4x), one fused the egg cell and the other one the central cell (apospory + double fertilisation; B_III_ formation pathway; Asker & Jerling, 1992; *c*. 7.4x endosperm; *c*. 4m : 1p contributions to the endosperm; Fig. 3e; Table 3). The variety of cytological pathways, gamete ploidies and relative parental contributions involved in seed formation demonstrates the diversification in functional reproductive strategies shown in polyploid *Ranunculus* hybrids.

**Table 2:** Flow cytometry seed analyses of functional pathways for seed formation in different *Ranunculus* hybrid types

Hybrid type	No. of individuals	No. of seeds	Seed class	Peak index Mean ± SD	Range of peak indexes	Maternal : paternal genomes in the endosperm^1^	Functional paths (%) ± SD
2x syn	15	501	Sex	1.55 ± 0.08	1.38–1.79	2m : 1p	100.0 ± 0.0
		–	Apo	–	–	–2	0.0 ± –
3x syn	10	58	Sex	1.64 ± 0.14	1.30–1.88	*c*. 2m : 1p	95.1 ± 7.6
		2	Apo	2.45 ± 0.05	2.42–2.49	*c*. 4m : 1p^3^	3.3 ± 5.6
		1	B_III_	1.69 ± –	–	*c*. 4m : 1p^3^	1.6 ± 0.0
6x nat	10	214	Sex	1.65 ± 0.13	1.48–1.99	2m : 1p	29.1 ± 12.4
		520	Apo	2.62 ± 0.21	2.33–3.07	2m : 1p or 4m : 1p	70.9 ± 12.4

syn, synthetic; nat, natural; Functional paths, effective reproductive pathways within each analysed hybrid type; SD, standard deviation of the sample or among genotypes; sex, sexual derived seeds; apo, asexual derived seeds; B_III_, seed derived from fertilisation of an unreduced embryo sac.

1 Genome contributions inferred from embryo sac structure, ploidies of embryo and endosperm tissues (Fig. 3; Table 3), expected gamete ploidies and observed chromosome numbers of F_2_ individuals (Supporting Information Table S6). Due to aneuploid gametes, only approximate genomic contributions are inferred for triploid individuals.

2 The expected ratio derived from an unreduced embryo sac would be 4m : 1p.

3 According to embryo ploidy and peak index, a near 1.5x male gamete has fertilised the polar nuclei.

**Table 3:** Ploidal variation observed in seed embryos according to ploidy, origin of genotypes and nature of their gametes

Materials	No. of seeds	Em ploidy mean 2*n* ± CV	Range 2*n*	CI	
				Lower	Upper
2x nat	30	2.01 ± 0.02	1.91–2.06	1.97	2.05
2x syn	501	2.02 ± 0.05	1.77–2.29	1.99	2.05
3x syn	61	2.81 ± 0.18	2.19–4.40^1^	2.62	3.03
6x nat	734	6.07 ± 0.05	5.66–6.28	5.98	6.15

nat, natural genotypes; syn, synthetic genotypes; Em, embryo; CI, confidence intervals; 2*n*, sporophytic ploidy level; CV, coefficient of variation; lower and upper bounds of 95% Confidence Intervals for Means.

1 Minimum value of near-diploid genotypes, maximum value representing a tetraploid genotype after partial apomixis.

Sown F_2_ seeds from triploid synthetic hybrids showed 24% germinability. Chromosomal counts on somatic cells of six surviving F_2_ plants revealed polyploidy in most individuals (Table S6).

## Discussion

The diversity of factors influencing natural history and reproductive behaviours of apomictic systems are becoming well-known in plant research (e.g. Ortiz *et al.* 2013; Hojsgaard *et al.*, 2014,). Although attributes responsible for the maintenance of apomicts in nature have been characterised (e.g. Paun *et al.* 2006b), the origin of new apomicts remains elusive. The emergence of aposporous-like processes among sexual hybridising species has not been previously evaluated and sheds new light on establishment of new apomicts.

### Ovule developmental features and activation of apomixis-like processes as an alternative reproductive pathway

Sexual reproduction is entrenched among flowering plants, the *Polygonum* type of female gametophyte development is prevalent (Maheshwari, 1950; Huang & Russell, 1992), and is characteristic of the diploid *Ranunculus notabilis*, *R. carpaticola* and autotetraploid *R. cassubicifolius* materials analysed here. Patterns of plant and ovule development are complex and controlled by a plethora of genes and epigenetic programs (Sieber *et al.* 2004; Grimanelli, 2012). In our studies, the standard sexual program in *Ranunculus* synthetic hybrids is clearly altered: on the one hand sporogenesis completion takes longer compared to parental species (see ovule rotation comparisons; Fig. S3, Table S2) and shows important developmental irregularities resulting in reduced fertility. These irregularities are associated with severe cytological abnormalities that constrain ovule and gamete development in synthetic hybrids by restricting the normal progression of meiosis and embryo sac formation (Fig. S4, Tables1, 3). Reductions in gamete viability and functionality (Tables1, S3, S4) further inhibit post-fertilisation seed development in diploid (≈20%) and triploid hybrids (< 1%; Table S5) (see further details in the next section). On the other hand, patterns of ovule development and gametogenesis in natural and synthetic hybrids show significant differences in timing and proportions of seed formation. When compared to synthetic hybrids, the sexual pathway for gamete formation in natural hybrids shows less radical alterations and at the same time displays higher frequencies of functional apospory (Tables1, 2, S3, S5). The delay in timing of sporogenesis is not as remarkable in natural hybrids as in synthetic hybrids (Fig. S2, Table S2).

Overall, the data suggest that hybridisation, rather than ploidy, is responsible for the ovule developmental alterations observed in *Ranunculus* hybrids. Such alterations of sexual reproductive programs in synthetic and natural hybrids are a probable consequence of disturbed intercommunication between signalling factors of merged divergent genomes, affecting the timing and scale of gene expression during development. Nonadditive and transgressive gene expression changes have already been documented in natural and resynthesized sexual hybrids (Adams, 2007; Hegarty *et al.* 2008). As gene expression changes can facilitate hybrid differentiation and speciation (e.g. *Senecio*; Hegarty *et al.* 2008), they also affect the relative fertility of hybrids by interfering with the normal development of reproductive traits. Furthermore, both synthetic and natural *Ranunculus* hybrid types show the appearance of apomixis or apomixis-like processes (Table 2). According to Carman (1997), interspecific hybridisation can desynchronize the timing of reproductive programs causing apomixis. Massively deregulated gene expression patterns in ovules of other facultative apomictic plants (Polegri *et al.* 2010; Sharbel *et al.* 2010) and in *Ranunculus auricomus* (M. Pellino *et al*., unpublished) support this idea, and are probably the main reason for the observed developmental alteration in *Ranunculus* hybrids. Thus, interspecific hybridisation in *Ranunculus* not only alters the sexual program, but also activates an alternative (asexual) pathway to seed formation. In this respect, together with the meiotic-associated reduction in fertility, there are concomitant changes in cell fate specification and cellular reprogramming in nucellar tissue of *Ranunculus* hybrids. Mutants of *Arabidopsis* for specific genes of the ARGONAUTE (AGO) family have shown the emergence of aposporous-like cells in the nucellus, suggesting that such genes are involved in cell specification changes during development (Grimanelli, 2012). In *Ranunculus* synthetic hybrids the reprogramming of nucellar cells into putative aposporous initials is significantly correlated with the arrested development and abortion of the germinal line. Such a change in cell fate specification of nucellar cells is also observed in natural hexaploid hybrids, and is correlated with arrested development of the meiotic pathway rather than ovule abortion. Our data suggest that interspecific hybridisation in *Ranunculus* could have a similar effect to that observed in *Arabidopsis* AGO mutants by altering expression patterns of heterologous AGO genes merged together in *Ranunculus* hybrid ovules. Such an effect could be responsible not only for the observed changes in nucellar cell destiny, but also for the observed correlation between the emergence of (putative) aposporous cells and megaspore abortion in synthetic and natural hybrids. Moreover, the lack of correlation between apomixis and ovule abortion and the more stable reproductive pathways in 60 000–80 000 yr old natural hybrids suggest that selective forces may act after hybridisation and new polyploid formation to control or rather stabilise developmental features (i.e. gene communication and expression patterns) in order to maintain reproductive functions (i.e. seed formation) that can overcome and/or even displace meiotic abnormalities (as seen in established natural hybrids).

### Balanced-unbalanced gametes, endosperm formation and seed set

The formation of reduced gametes is crucial to keep maternal-to-paternal dosages required for post-fertilisation seed development in many angiosperms. Deviations from 2m : 1p (two polar nuclei to one sperm) genomic contributions cause aberrant endosperm development whereby overdoses in genomic contributions of any parental have drastic effects on embryo and endosperm sizes (e.g. Lin, 1984; Scott *et al.* 1998; Kradolfer *et al.* 2013). Sexual diploid and tetraploid *R. auricomus* are sensitive to endosperm imbalance (Rutishauser, 1954; Hörandl & Temsch, 2009). Despite the altered developmental pattern, synthetic diploid *Ranunculus* hybrids formed reduced (haploid) embryo sacs and pollen, which guaranteed appropriate maternal-to-paternal genome contributions after cross-fertilisation. Hence, differences between observed functional embryo sacs and seed set levels are probably due to constraints related to genetic divergence between the hybridising species.

Conversely, cytological observations, ploidies and genomic ratios of embryos to endosperms on seeds indicate that synthetic triploids produce an array of gametes with different chromosome numbers as a consequence of disturbed meiosis (Tables2, 3, S6). An excess or deficiency of specific chromosomes caused by aneuploidy can generate gene dosage imbalance with negative consequences on gametophytes (Henry *et al.* 2005, 2007), and dramatic reduction in fertility of sexual triploid plants (Khush, 1973). Beside genomic divergence between the hybridised species, gametic aneuploidy in *Ranunculus* synthetic triploids is a major factor for the exceptionally reduced fertility, causing (1) low gamete viability and (2) functional disability (both discussed in the previous part of the Discussion), and (3) genomic unbalances to embryo and endosperm formation. Peak index ratios in F_1_ triploid hybrids show that sexual seed formation was driven by the ploidy (chromosome number) of the female gametophyte, as only male gametes that balanced the ploidy of female gametes and hence contribute *c*. 2m : 1p genomes to the endosperm were effective (Table 2). Similar observations were found in other triploid experimental systems (e.g. *Paspalum* spp., Martínez *et al.*, 2007). Nonetheless, along with sexuality, *Ranunculus* synthetic triploids exhibit other patterns of seed formation (i.e. partial apomixis, functional apomixis). Seed development in apomicts suffers from gametic ploidy asymmetry (unreduced embryo sacs and reduced sperms) and generates an endosperm-balance problem as the 2m : 1p balance is shifted to 4m : 1p (Talent & Dickinson, 2007). Natural apomicts have developed various modifications to restore the 2m : 1p balance, as in *Panicum*, *Dichantium* and *Crataegus* (Reddy & D'Cruz, 1969; Nogler, 1984; Talent & Dickinson, 2007), but in general apomictic seeds tolerate deviations in parental genome contributions, from 2m : 1p till 8m : 1p (e.g. *Paspalum* spp., Quarin, 1999; *Potentilla* spp., Dobeš *et al.*, 2013). In our synthetic triploids, seeds derived from unreduced embryo sacs had endosperm contributions of *c*. 4m : 1p genomes (Table 2). In the same way, sexual seeds from reduced gametes in natural established hexaploid *Ranunculus* maintain the 2m : 1p balance, but apomictic seeds showed shifts in endosperm contributions (to 4m : 1p) which was partially restored to 2m : 1p by the fusion of two sperms to the central cell (Fig. 3f, Table 2). Altogether, the otherwise strict dosage balance required for endosperm development in the sexual pathway is relaxed in the apomictic one. Whether this relaxation is due to apomictic embryo formation or to ploidy effects, however, needs to be studied. In synthetic triploids, the absence of balanced genome contributions caused by aneuploidy is probably responsible for the failure of sexual seed development, but at the same time represents a persistent pressure on the reproductive system to retain seed formation capacities by selecting for gametes/factors that restore such stoichiometric balances or rather tolerate deviations. Hence, even low rates of functional apomixis would be favoured to functional sexuality in odd polyploid genotypes with high rates of unbalanced gametes, as asexual polyploid seeds escape endosperm-balance restrictions and can potentially use the whole array of produced male gametes.

### Establishment of new apomicts

To summarise, hybridisation seems to trigger the emergence of apomixis as a consequence of asynchronous development in the *Ranunculus auricomus* aggregate. Polyploidy combined with self-compatibility would facilitate the selection of functional apomixis due to permissive conditions to endosperm formation, but also as a factor stabilising the genetic constitution of the (parthenogenetic) embryo.

Likewise, our results suggest that apomixis components are still uncoupled in synthetic hybrids. Selection against partial apomixis (van Dijk & Vijverberg, 2005) can promote the developmental stabilisation of the asexual pathway and herewith the establishment of small polyploid apomictic populations. In this respect, apomixis in tetraploid *Paspalum malacophyllum* is highly unstable during sporogenesis and at the beginning of gametogenesis, but becomes stabilised by the end of gametogenesis (Hojsgaard *et al.* 2013), a possible signature of past instabilities during establishment of functional apomixis in the species. Natural hexaploid 60 000–80 000 yr old *Ranunculus* hybrids exhibit intermediate ovule developmental characteristics between sexual parentals and synthetic hybrids plus both apomictic-permissive and sexual-restrictive pathways to endosperm formation. Selection for the stabilisation of developmental features is supported by transcriptome-wide single nucleotide polymorphism (SNP) analyses (Pellino *et al.* 2013) that revealed evidence for natural diversifying selection acting on an enrichment of genes involving different meiotic steps subsequent to the divergence of *Ranunculus* natural hybrids from sexual parental species.

### Conclusion

Interspecific hybridisation can alter nucellar cell specification to trigger apospory via asynchronous developmental patterns. Developmental alterations associated with sexual seed formation are less extreme in diploid than in triploid synthetic hybrids. Levels of female and male gamete viability and correlation among functional reproductive states suggest that both reproductive pathways are interrelated but not yet balanced in synthetic triploids as in the natural hexaploid hybrids. Polyploidy is a key factor for functional apomixis, as it allows for selection of pathways tolerating deviations on paternal-to-maternal genome contributions in the endosperm and successful seed formation. Moreover, as partial apomixis rises up ploidies, it may play a temporal role by shifting from genetically and chromosomally unstable odd-ploidal genotypes to more stable even-ploidal genotypes. Thus, along an evolutionary timescale, apomixis would be selectively favoured together with factors promoting its coexistence with the meiotic reproductive pathway during the establishment of new polyploid apomictic lineages.

## Supporting Information

Fig S1 Key reproductive stages (i.e. end of female meiosis, selected functional megaspore, and mature embryo sac) during flower development of sexual species from *Ranunculus auricomus* complex. **Fig. S2** Results supporting the hybrid nature of progenies from homo- and heteroploid interspecific crossings. **Fig. S3** Ovule rotation images of complete ovaries at two key developmental stages (end of meiosis and end of gametogenesis) for parental species, natural and synthetic hybrids and dispersion plot for all measured angles in parental, hexaploid and synthetic hybrid genotypes. **Fig. S4** Images depicting different ovule developmental irregularities observed among synthetic hybrids. **Table S1** List of wild sexual (including parental genotypes) and natural hybrid materials used for the reproductive characterization and to generate the synthetic hybrid populations **Table S2** Variation in ovule rotation angles observed in sexual parental, natural hybrids and synthetic hybrids **Table S3** Analyses of male gamete viability through pollen stainability measures in parental genotypes (*R. notabilis*, *R. carpaticola* and *R. cassubicifolius*), natural and synthetic hybrids **Table S4** Time intervals for pollen tube development through pistil tissues in sexual parental genotypes, synthetic and natural hybrids of *R. auricomus* complex **Table S5** Seed production analyses in parental genotypes (*R. notabilis*, *R. carpaticola* and *R. cassubicifolius*), natural and synthetic hybrids **Table S6** Ploidy levels and parental contribution to F_2_ progenies from crosses between triploid (3x) *R. cassubicifolius* × *R. notabilis* genotypes
