# Supplementary material for: Emergence of apospory and bypass of meiosis via apomixis after sexual hybridisation and polyploidisation
Source: New Phytol. 2014 Jul 31;204(4):1000–12. doi: 10.1111/nph.12954 (PMC4260133; doi:10.1111/nph.12954)
Supplement: Fig S1 — Key reproductive stages (i.e. end of female meiosis, selected functional megaspore, and mature embryo sac) during flower development of sexual species from Ranunculus auricomus complex. Fig. S2 Results supporting the hybrid nature of progenies from homo- and heteroploid interspecific crossings. Fig. S3 Ovule rotation images of complete ovaries at two key developmental stages (end of meiosis and end of gametogenesis) for parental species, natural and synthetic hybrids and dispersion plot for all measured angles in parental, hexaploid and synthetic hybrid genotypes. Fig. S4 Images depicting different ovule developmental irregularities observed among synthetic hybrids. Table S1 List of wild sexual (including parental genotypes) and natural hybrid materials used for the reproductive characterization and to generate the synthetic hybrid populations Table S2 Variation in ovule rotation angles observed in sexual parental, natural hybrids and synthetic hybrids Table S3 Analyses of male gamete viability through pollen stainability measures in parental genotypes (R. notabilis, R. carpaticola and R. cassubicifolius), natural and synthetic hybrids Table S4 Time intervals for pollen tube development through pistil tissues in sexual parental genotypes, synthetic and natural hybrids of R. auricomus complex Table S5 Seed production analyses in parental genotypes (R. notabilis, R. carpaticola and R. cassubicifolius), natural and synthetic hybrids Table S6 Ploidy levels and parental contribution to F2 progenies from crosses between triploid (3x) R. cassubicifolius × R. notabilis genotypes [file nph0204-1000-SD1.docx]

## *New Phytologist* Supporting Information

**Emergence of apospory and bypass of meiosis via apomixis after sexual hybridisation and polyploidisation**

Diego Hojsgaard, Johann Greilhuber, Marco Pellino, Ovidiu Paun, Timothy F. Sharbel and Elvira Hörandl

Article acceptance date: 24 June 2014

The following Supporting Information is available for this article:

**
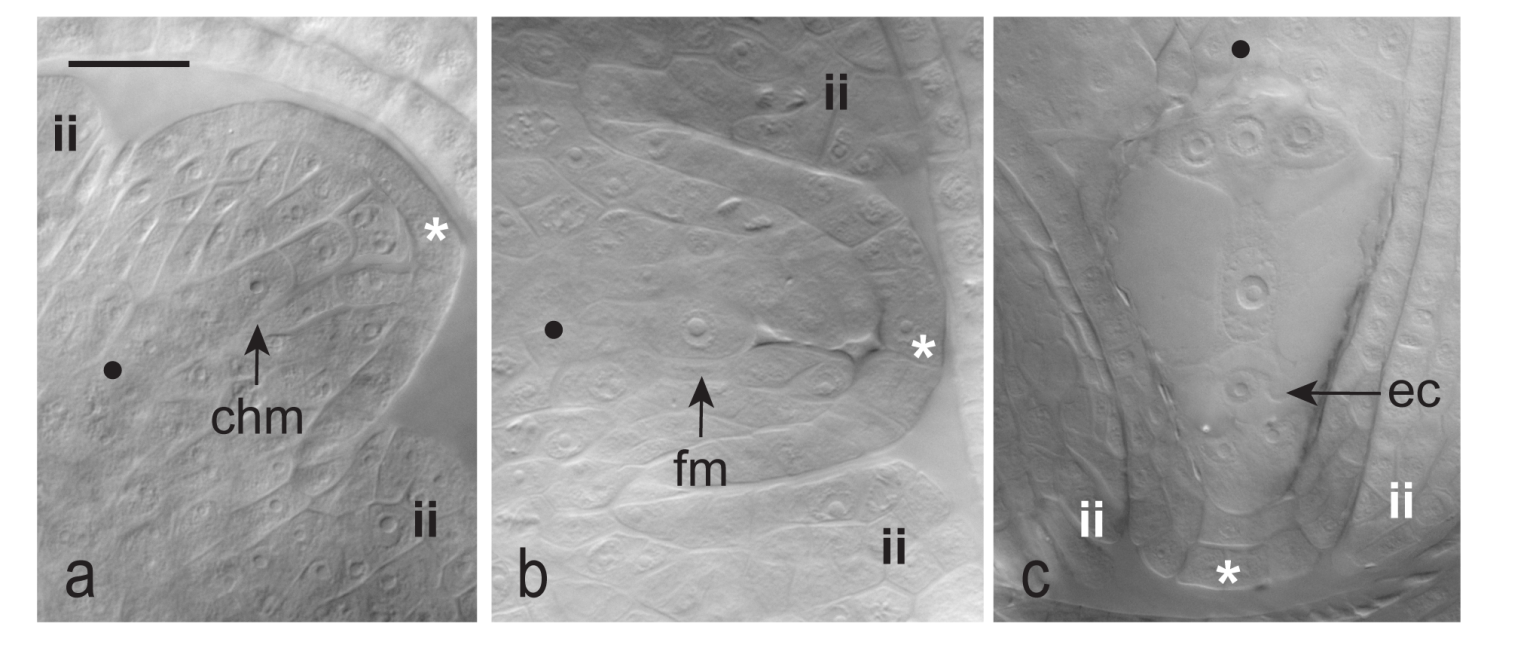
 Fig. S1** Key reproductive stages during flower development of sexual species from *Ranunculus auricomus* complex. Each image shows an unitegmic ovule with the main growing axis perpendicular to the surface of the flower head receptacle (here represented parallel to the base of every image). Hence, the ovule rotation can be followed (for details see Fig. S3). **a:** end of meiosis showing three functional megaspore cells, the micropylar cell remains binucleated as cytokinesis after telophase II does not occur; **b:** selected functional megaspore and degenerated sister micropylar cells; **c:** completely rotated ovule showing a mature embryo sac at blooming stage (egg and synergid cells are rooted at the micropylar area). **a** and **b:** 2x *R. carpaticola* Hö8483; **c:** 2x *R. notabilis* Hö5612. **chm:** chalazal megaspore; **ec:** egg cell; **fm:** functional megaspore; **ii:** inner integuments; **•:**chalazal pole; ***:** micropylar pole. Bar represents 30 µm.

**
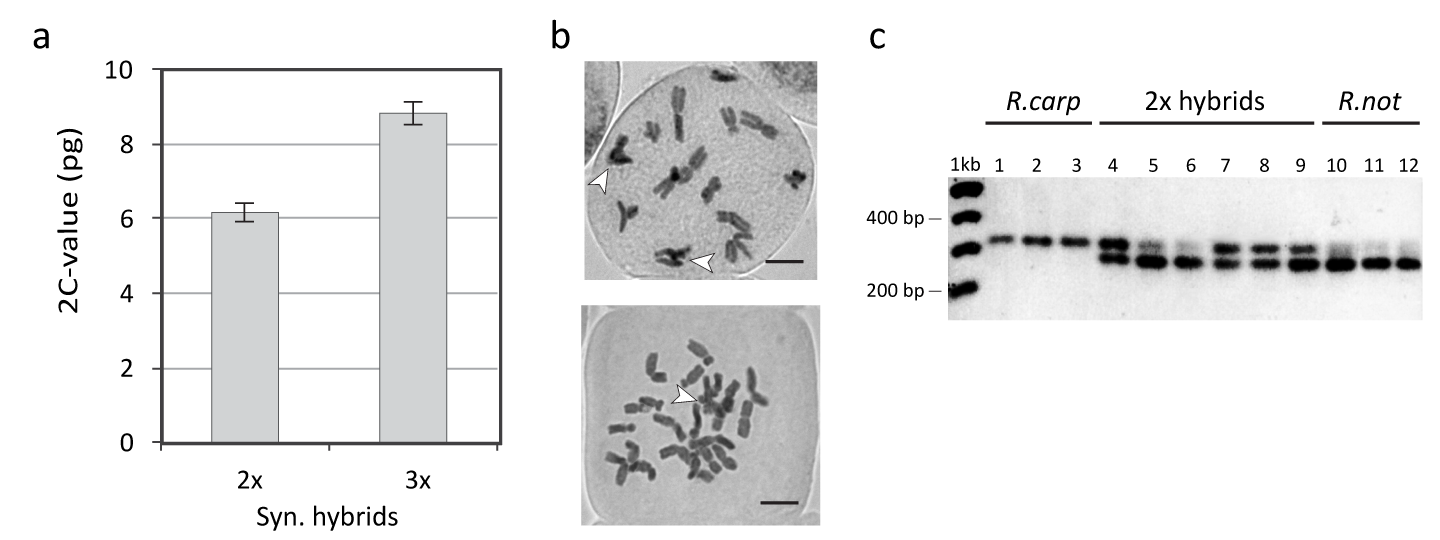
**

**Fig. S2** Results supporting the hybrid nature of progenies from homo- and heteroploid interspecific crossings. **a:** Histograms for absolute DNA content of 43 diploids *R. carpaticola* × *R. notabilis* (2C=6.20 pg ± 0.26) and 39 triploids *R. cassubicifolius* × *R. notabilis* progeny (2C=8.84 pg ± 0.31) (see also Table S1); **b:** selected images of mitotic metaphase cells confirming 2n=2x=16 (from genotype J25) and 2n=3x=24 (from genotype G2) chromosome complements for diploid and triploid synthetic individuals, respectively; **c:** pattern of microsatellite segregation between parental genotypes (*R. carpaticola* and *R. notabilis*) and their diploid hybrids showing a paternally inherited allele present in the male parent (lines 10-12) and its progeny. Primer pair *Rau*10 amplify an allele including a (TTC)_3_(TCTTC)_2_ repetition absent in female parents; in few genotypes extra variation was obtained from a (GA)_7_ repetition placed at *Rau*10 SSR flanking region. Line 1-3 represents maternal *R. carpaticola* genotypes (Hö8483). Lines 4-9 represent diploid hybrid genotypes J1, J2, J3, J4, J37 and a bulk of 13 different diploid hybrids, respectively. Lines 10-12 represent paternal *R. notabilis* genotypes (for details see Table S1). Whiskers in **a** represent 95% CI; arrows in **b** indicate two overlapping chromosomes; *R. carp*= *R. carpaticola*; *R. not*= *R. notabilis*; 1kb: 1kb ladder (Invitrogen). Bars represent 5 µm.

**
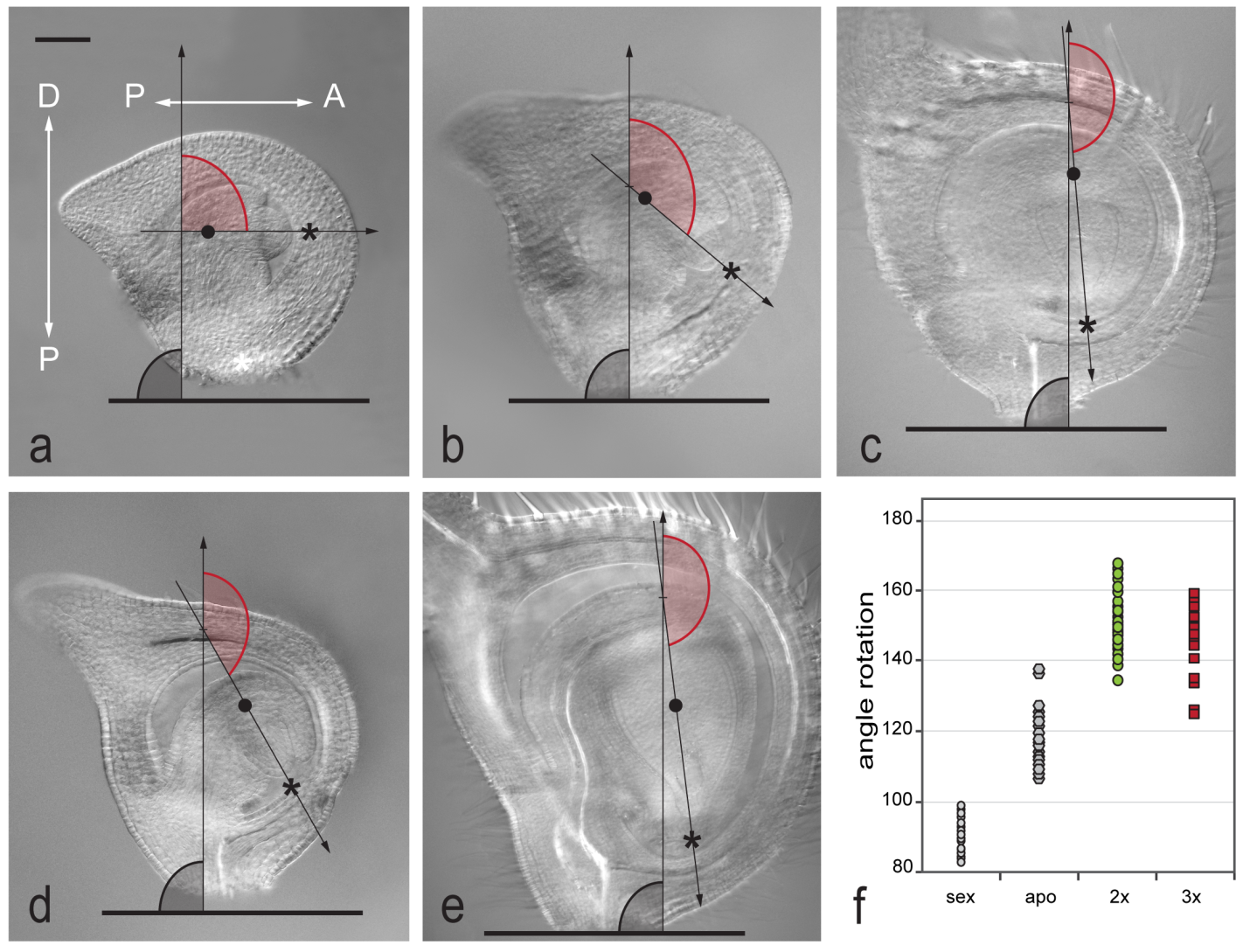
**

**Fig. S3** Images of complete ovaries at two key developmental stages: end of meiosis and end of gametogenesis (flowering). All ovaries were placed with the same spatial orientation and perpendicular (dark shadowed angle) to the receptacle of the flower head, here represented by the basal line (thick line). The two main axes of growth were defined (P-D; A-P). For every single ovary, an imaginary line was traced, going through the main axis of the linear tetrad or the mature embryo sac from the chalazal (dot) to the micropylar pole (asterisk). Then, the ovule rotation (red shadowed angle) was measured as the angle between this imaginary line and the P-D axis of the ovule. **a:** diploid *R. carpaticola* (Hö8483) at the end of meiosis; **b:** hexaploid *R. carpaticola* × *R. cassubicifolius* genotype (HöC29) at the end of meiosis; **c:** diploid *R. notabilis* (Hö5612) at the end of gametogenesis; **d:** triploid *R. cassubicifolius* × *R. notabilis* genotype (G6) at the end of meiosis; **e:** triploid *R. cassubicifolius* × *R. notabilis* genotype (G13) at the end of gametogenesis; **f:** Dispersion plot for all measured angles in parental genotypes (sex), hexaploid genotypes (apo), and synthetic hybrid genotypes (2x, 3x) (for details see Table S2). P-D: proximal-distal axis; A-P: anterior-posterior axis. Bar represents 150 µm.


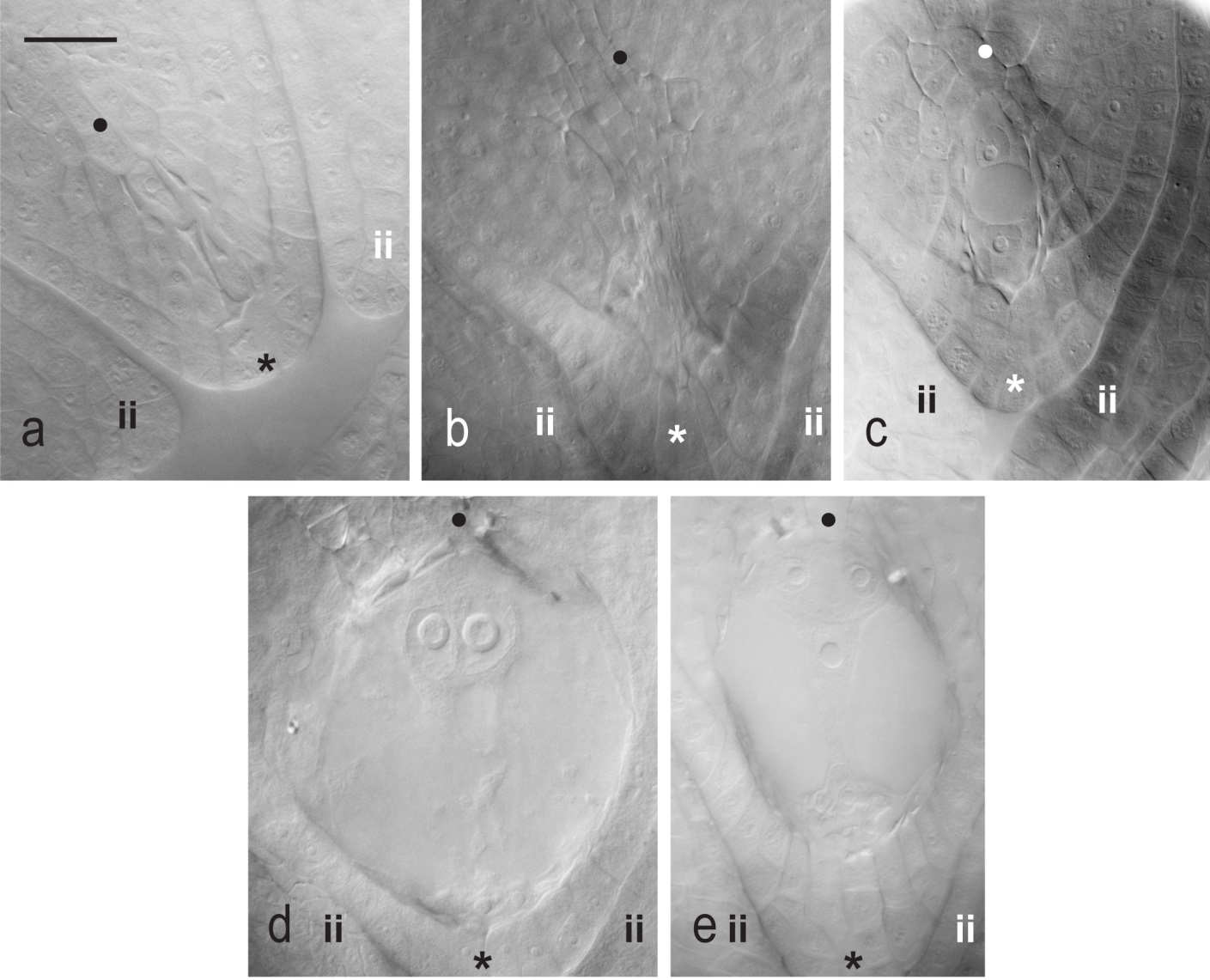


**Fig. S4.** Images depicting different ovule developmental irregularities observed among synthetic hybrids. Ovules are placed as in Fig. S1, hence ovule rotation can be tracked. **a:** end of meiosis showing a functional megaspore with sign of abortion; **b:** ovule at flowering stage without embryo sac; **c:** female gametophyte at 2-nuclei stage depicting a delayed or arrested development of the gametogenesis program; **d:** female gametophyte at flowering having all syncytial nuclei at the micropylar pole aborted and abnormal growth of nuclei at the chalazal pole; **e:** mature female gametophyte with all micropylar cells and polar nucleus aborted while cells from the chalazal pole including the chalazal polar nucleus remain functional. **a:** J20; **b:** J10; **c:** G6; **d:** G5; **e:** I5. **ii:** inner integuments; **•:**chalazal pole; ***:** micropylar pole. Bar represents 30 µm.

**Table S1** List of wild sexual (including parental genotypes) and natural hybrid materials used for the reproductive characterization and to generate the synthetic hybrid populations (see also Fig. S1)

| Taxa | Accession ID  (n° of individuals) | Chromosome numbers (Ploidy) | 2C-value (pg) § | CV (%) | Voucher (herbarium ¤) |
| --- | --- | --- | --- | --- | --- |
| ***Parentals*** |  |  |  |  |  |
| *R. notabilis* | 7224* (1); 9609* (6) | 16 (2x)† | nd |  | *Hörandl 5612* (WU) |
| *R. carpaticola* | 8483* (6); 8486(10) | 16 (2x)‡ | 6.12 | 4.1 | *Hörandl 8483* (WU); *Hörandl 8486* (WU) |
| *R. cassubicifolius* | 8472* (2); 8473* (3) | 32 (4x)‡ | 11.37 | 4.2 | *Hörandl 8472* (WU); *Hörandl 8473* (WU) |
| ***Experimental hybrids*** |  |  |  |  |  |
| *R. carpaticola* × *R. notabilis* ^1^ | F(12); J(38) | 16 (2x) | 6.20 | 4.2 | --- |
| *R. cassubicifolius* × *R.* *notabilis* ^2^ | G (22); H(5); I(5) | 24 (3x) | 8.84 | 3.5 | --- |
| ***Natural hybrids*** |  |  |  |  |  |
| *R. carpaticola* × *R. cassubicifolius* | 8492/6(12); 8492/27(22); 29/15(30); 35/28(37) | 48 (6x)† | 16.1 | 3.3 | *Mládenkova, Paun & Hörandl C29* (SAV); *Mládenkova, Paun & Hörandl C35* (SAV); *Mládenkova, Paun & Hörandl C41* (SAV); *Hörandl 8492* (WU) |

nd= non determined; CV(%)= absolute values of the coefficient of variation.

^1^ F and J genotypes are derived from 8483 × 7224 crosses

^2^ G individuals are derived from 8472 × 9609 crosses; H and I are derived from 8473 × 9609 crosses

* parental genotypes in experimental hand-made pollinations

†Hörandl *et al.* 2000

‡Hörandl & Greilhuber 2002; Paun *et al.* 2006

§DNA content of non-replicated holoploid non-reduced genomes (after Greilhuber *et al.* 2005).

¤ Herbarium codes according to Index Herbariorum (<http://sciweb.nybg.org/science2/IndexHerbariorum.asp>)

**Greilhuber J. 2005.** The origin, evolution and proposed stabilization of the terms 'genome size' and 'C-value' to describe nuclear DNA contents. *Annals of Botany* (London) **95:** 255-260.

**Hörandl E, Greilhuber J, Dobeš C. 2000.** Isozyme variation within the apomictic *Ranunculus auricomus* complex: evidence for a sexual progenitor species in southeastern Austria. *Plant Biology* **2:** 1-10.

**Hörandl E, Greilhuber J. 2002.** Diploid and autotetraploid sexuals and their relationships to apomicts in the *Ranunculus cassubicus* group: insights from DNA content and isozyme variation. *Plant Systematic and Evolution* **234:** 85-100.

**Paun O, Stuessy TF, Hörandl E. 2006.** The role of hybridization, polyploidization and glaciation in the origin and evolution of the apomictic *Ranunculus cassubicus* complex. *New Phytologist* **171:** 223-236.

**Table S2** Variation of ovule rotation observed in sexual parental, natural hybrids and synthetic hybrids (see Fig. S3). Confidence intervals are giving for an α=0.05

| Developmental stage | *n* | Angle rotation*  ± SD | Var | Range | C I |
| --- | --- | --- | --- | --- | --- |
|  |  |  |  |  | lower upper |
| ***End of meiosis*** |  |  |  |  |  |
| Sexual parental genotypes | 17 | 93.24 ± 5.27 | 27.79 | 84.60 – 100.83 | 90.73 – 95.74 |
| Synthetic hybrids |  |  |  |  |  |
| Diploids | 45 | 151.47 ± 7.49 | 56.06 | 134.07 – 167.03 | 149.28 – 153.65 |
| Triploids | 23 | 148.35 ± 9.82 | 96.52 | 125.13 – 159.63 | 144.33 – 152.36 |
| Natural hybrids | 22 | 119.23 ± 7.81 | 60.96 | 107.79 – 137.27 | 115.65 – 122.49 |
| ***Flowering*** |  |  |  |  |  |
| Sexual parental genotypes | 15 | 165.36 ± 2.11 | 4.46 | 162.70 – 169.09 | 164.29 – 166.46 |
| Synthetic hybrids |  |  |  |  |  |
| Diploids | 22 | 163.72 ± 6.68 | 44.56 | 146.39 – 172.78 | 160.93 – 166.51 |
| Triploids | 26 | 168.90 ± 4.64 | 21.56 | 162.15 – 178.04 | 167.12 – 170.69 |
| Natural hybrids | 16 | 165.33 ± 4.04 | 16.34 | 156.30 – 171.24 | 163.35 – 167.31 |

* sexagesimal angles were converted to decimal degrees (deg) by using the following transformation: degrees as unit, minutes/60 + seconds/3600 were added as decimals.

*n*= number of measured ovules; SD= standard deviation of the sample; Var= variance of sampled ovule rotation values; CI= 95% confidence intervals.

**Table S3** Analyses of male gamete viability through pollen stainability measures in parental genotypes (*R. notabilis*, *R. carpaticola* and *R. cassubicifolius*), natural and synthetic hybrids

|  | Ni | Pollen grains | Viability  Mean ± SEM | Min | Max | Var | C I | |
| --- | --- | --- | --- | --- | --- | --- | --- | --- |
| Materials |  |  |  |  |  |  | lower | upper |
| ***Parental genotypes*** | 6 | 1773 | 79.2 ± 4.89 | 66.0 | 94.1 | 143.37 | 66.64 | 91.77 |
| ***Synt. hybrids*** |  |  |  |  |  |  |  |  |
| **J** and **F** ^1^ | 12 | 6198 | 45.3 ± 2.78 | 32.3 | 62.4 | 84.84 | 35.71 | 56.32 |
| **G** and **I** ^2^ | 9 | 5342 | 18.3 ± 1.54 | 10.6 | 25.9 | 21.30 | 14.41 | 23.93 |
| ***Natural hybrids*** | 6 | 4199 | 53.2 ± 4.62 | 34.0 | 74.7 | 192.29 | 33.54 | 65.05 |

^1^ F and J genotypes are derived from 8483 × 7224 crosses (details of genotype codes in Table S1)

^2^ G individuals are derived from 8472 × 9609 crosses; H and I are derived from 8473 × 9609 crosses Ni= number of individuals studied; Pollen grains= number of male gametophytes analyzed; SEM= standard error of the mean; Var= variance; lower and upper bounds of 95% Confidence Intervals (CI) for Means (α=0.05).

**Table S4** Time intervals for pollen tube development through pistil’s tissues in sexual parental genotypes, synthetic and natural hybrids of *R. auricomus* complex

|  |  | Pollen tubes at | | | |
| --- | --- | --- | --- | --- | --- |
| Pistiled flower × pollen donor*  (ploidies) | n | stigma | style | ovary | micropyle |
| ***Parentals*** |  |  |  |  |  |
| *R. not* × *R. not*  (2x × 2x) | 27 |  |  |  |  |
| 30min |  | +++ |  |  |  |
| 60min |  |  | +++ | + |  |
| 120min |  |  |  | ++ | + |
| 180min |  |  |  | ++ | + |
| *R. cass* × *R. cass*  (4x × 4x) | 20 |  |  |  |  |
| 30min |  | +++ |  |  |  |
| 60min |  |  | ++ | + |  |
| 120min |  |  |  | + |  |
| 180min |  |  |  | + | + |
| ***Synthetic hybrids*** |  |  |  |  |  |
| *R. carp* × *R. not*  (2x × 2x) |  |  |  |  |  |
| **J × J** | 16 |  |  |  |  |
| 30min |  | +++ |  |  |  |
| 60min |  |  | +++ | + |  |
| 120min |  |  |  | ++ |  |
| 180min |  |  |  | + | + |
| **J × F** or **F × J** | 40 |  |  |  |  |
| 30min |  | +++ |  |  |  |
| 60min |  |  | +++ | + |  |
| 120min |  |  |  | ++ |  |
| 180min |  |  |  | + | + |
| **F × F** | 17 |  |  |  |  |
| 30min |  | +++ |  |  |  |
| 60min |  |  | +++ |  |  |
| 120min |  |  |  | + |  |
| 180min |  |  |  | + | + |
| *R. cass* × *R. not*  (3x × 3x) |  |  |  |  |  |
| **G × G** | 30 |  |  |  |  |
| 30min |  | + |  |  |  |
| 60min |  |  | + |  |  |
| 180min |  |  |  | + | + |
| **G × I** or **I × G** | 14 |  |  |  |  |
| 30min |  | ++ |  |  |  |
| 60min |  |  | ++ |  |  |
| 180min |  |  |  | + | + |
| **I × I** | 20 |  |  |  |  |
| 30min |  | ++ |  |  |  |
| 60min |  |  | ++ |  |  |
| 180min |  |  |  | + | + |
| ***Natural hybrids*** |  |  |  |  |  |
| *R. carp* × *R.* *cass*  (6x × 6x) | 38 |  |  |  |  |
| 30min |  | +++ |  |  |  |
| 60min |  |  | +++ |  |  |
| 120min |  |  |  | ++ |  |
| 180min |  |  |  | + | + |

*R. not*= *Ranunculus notabilis*; *R. cass*= *R. cassubicifolius*; *R. carp*= *R. carpaticola*; *R. carp* × *cass*= *R. carpaticol* × *cassubicifolius* natural hybrid, F and J genotypes are derived from 8483 × 7224 crosses; G individuals are derived from 8472 × 9609 crosses; H and I are derived from 8473 × 9609 crosses; n=number of ovaries analysed;

* for details on genotypes codes see Table S1.

+++= 10 or more pollen tubes observed;

++= 3-9 pollen tubes;

+= 1-2 pollen tubes.

**Table S5** Seed production analyses in parental genotypes (*R. notabilis*, *R. carpaticola* and *R. cassubicifolius*), natural and synthetic hybrids

|  | *N* | Mean | SEM | SD | Min | Max | Var | C I | | G-H *p* |
| --- | --- | --- | --- | --- | --- | --- | --- | --- | --- | --- |
| Groups |  | (%) |  |  |  |  |  | lower | upper |  |
| ***Parental genotypes*** | 27 | 78.02 | ± 2.67 | ± 13.9 | 55.8 | 98.0 | 193.03 | 72.53 | 83.52 | <0.01 |
| ***Synthetic hybrids*** |  |  |  |  |  |  |  |  |  |  |
| **J** and **F** | 46 | 20.68 | ± 2.74 | ± 18.6 | 0.0 | 76.7 | 345.56 | 15.16 | 26.20 | <0.01 |
| **G** and **I** | 29 | 0.86 | ± 0.37 | ± 2.0 | 0.0 | 7.9 | 3.90 | 0.11 | 1.61 | <0.01 |
| ***Natural hybrids*** | 26 | 45.68 | ± 2.61 | ± 13.3 | 30.1 | 75.3 | 177.14 | 40.30 | 51.06 | <0.01 |

*N*= number of individuals studied; SEM= standard error of the mean; SD= standard deviation; Var= variance; lower and upper bounds of 95% Confidence Intervals (CI) for Means; G-H *p*= post hoc Games-Howell test for Mean comparisons among groups when Homogeneity of Variances is violated (Levene´s test *p*<0.001).

**Table S6** Ploidy levels and parental contribution to F_2_ progenies from crosses between triploid (3x) *R. cassubicifolius* × *R. notabilis* genotypes

| **F_2_ progeny*** | Chr. number | | Ploidy | Ploidy of parental gametes† | | Chr. number of gametes‡ | |
| --- | --- | --- | --- | --- | --- | --- | --- |
|  | (2n) |  | | sexual | apomictic | sexual | apomictic |
| G1 × G9 | 32 | 4x | | ̴2x | 3x + x | 16 + 16 | 24 + 8 |
| G1 × G9 | 30 | 4x-2 | | ̴2x-1 | n.v. | 15 + 15 | n.v. |
| G19 × G9 | 24 | 3x | | 1.5x | 3x | 12 + 12 | 24 |
| G9 × G19 | 18 | 2x+2 | | ̴1.1x | n.v. | 9 + 9 | n.v. |
| G12 × G7 | ̴38-40 | 5x(-2) | | ̴2.5x | 3x + ̴2x | 19 + ̴20 | 24 + ̴16 |
| G16 × I2 | 30 | 4x-2 | | ̴2x-1 | n.v. | 15 + 15 | n.v. |

*the maternal genotype is placed to the left in each F_2_ progeny cross; Chr.= chromosome; †expected ploidies according to flow cytometry seed analyses, reproductive pathways and likelihood of viable chromosomal numbers (e.g. numbers below x are expected to be lethal); ‡expected chromosome numbers according to gamete ploidy; n.v.= non-viable
